# A physiologically based pharmacokinetic model of cefepime to predict its pharmacokinetics in healthy, pediatric and disease populations

**DOI:** 10.1016/j.jsps.2023.06.008

**Published:** 2023-06-19

**Authors:** Muhammad Talha Zahid, Ammara Zamir, Abdul Majeed, Imran Imran, Sary Alsanea, Tanveer Ahmad, Faleh Alqahtani, Muhammad Fawad Rasool

**Affiliations:** a Department of Pharmacy Practice, Faculty of Pharmacy, Bahauddin Zakariya University, 60800, Multan, Pakistan; b Department of Pharmaceutics, Faculty of Pharmacy, Bahauddin Zakariya University, 60800, Multan, Pakistan; c Department of Pharmacology, Faculty of Pharmacy, Bahauddin Zakariya University, 60800, Multan, Pakistan; d Department of Pharmacology and Toxicology, College of Pharmacy, King Saud University, Riyadh 11451, Saudi Arabia; e Institute for Advanced Biosciences (IAB), CNRS UMR5309, INSERM U1209, Grenoble Alpes University, La Tronche 38700, France

**Keywords:** Cefepime, Pharmacokinetics, Cephalosporin, Pediatrics, Chronic kidney disease, Obese, PBPK

## Abstract

The physiologically based pharmacokinetic modeling (PBPK) approach can predict drug pharmacokinetics (PK) by combining changes in blood flow and pathophysiological alterations for developing drug-disease models. Cefepime hydrochloride is a parenteral cephalosporin that is used to treat pneumonia, sepsis, and febrile neutropenia, among other things. The current study sought to identify the factors that impact cefepime pharmacokinetics (PK) following dosing in healthy, diseased (CKD and obese), and pediatric populations. For model construction and simulation, the modeling tool PK-SIM was utilized. Estimating cefepime PK following intravenous (IV) application in healthy subjects served as the primary step in the model-building procedure. The prediction of cefepime PK in chronic kidney disease (CKD) and obese populations were performed after the integration of the relevant pathophysiological changes. Visual predictive checks and a comparison of the observed and predicted values of the PK parameters were used to verify the developed model. The results of the PK parameters were consistent with the reported clinical data in healthy subjects. The developed PBPK model successfully predicted cefepime PK as observed from the ratio of the observed and predicted PK parameters as they were within a two-fold error range.

## 1 Introduction

Cefepime hydrochloride is an antibiotic with broad-spectrum activity against a variety of gram-positive and gram-negative microorganisms (Pais et al., 2022, Pais et al., 2022). In 1996, the United States Food and Drug Administration (FDA) authorized the use of cefepime as a treatment option for various infections that include urinary tract infections both complicated and uncomplicated, pneumonia, skin and soft tissue infections (Kim et al., 2010). Cefepime works by blocking the transpeptidases enzyme, which disrupts the creation of peptidoglycan, a crucial element of the bacterial cell wall. It is administered intravenously (IV) or intramuscularly (I.M) and is available in three different strengths: 500 mg, 1000 mg, and 2000 m (Authority).

Cefepime primarily attaches to albumin with an unbound fraction (fu) of 61.4 % in plasma (Al-Shaer et al., 2020). It exhibits linear kinetics following IV or I.M administration in both healthy and sick individuals (Van Der Auwera and Santella 1993). In individuals with normal kidney function its volume of distribution at steady state (Vss) is ≈ 0.22 L/Kg and half-life (t_1/2_) is 2–2.3 h (Van der Auwera and Santella 1993). The majority of the drug is excreted unchanged through urine with renal clearance (CL_R_) varying between 96 and 116 mL/min, while a small portion of the administered dose about (10–20%), undergoes metabolism to form a metabolite called N-methyl pyrrolidine (NMP). NMP is rapidly transformed to NMP-N-oxide (Van der Auwera and Santella, 1993, Endimiani et al., 2008).

Cefipime has a molecular weight of 517 g/mol and is water-soluble (hydrochloride). Moreover, its lipophilicity (LogP) is 0.6914 (AboulMagd et al., 2021). Cefepime belongs to the Biopharmaceutics Classification System (BCS) class 3. This means that it has high solubility, it can easily dissolve in fluids, but limited permeability, which means that it may have difficulty passing through certain barriers in the body, such as cell membrane this is particularly related to oral form of cefepime (Albenayan et al., 2022). Cefepime falls under pregnancy category B and is considered to be a safe drug during gestation (Authority). When cefepime is administered intravenously, it exhibits a high capacity to traverse the blood–brain barrier (BBB). This property makes cefepime an attractive option for treating infections of the CNS, such as meningitis and encephalitis, caused by susceptible microorganisms (Sullins and Abdel-Rahman 2013). However, it should not be used in patients with a history of hypersensitivity to the drug or other cephalosporin and beta-lactam antibiotics (Authority). Some of the adverse drug reactions associated with cefepime use include the development of a rash, which may appear as redness, itching, or hives on the skin. (Castle 2007). In addition, cefepime may cause phlebitis, which is the inflammation or swelling of a vein, which can lead to discomfort, redness, and warmth around the affected area (Gutierrez 2004).

Teorell introduced the world to the concept of PBPK in 1937 as it merges physiological and biological components with traditional mammillary models to provide a more comprehensive analysis of PK data (Teorell, 1937, Jones and Rowland-Yeo, 2013). PBPK modeling is becoming increasingly popular in drug discovery, development, and regulatory reviews (Zhuang and Lu 2016). The use of PBPK modeling is especially relevant in pediatrics as dosing in this population group presents many challenges (Johnson et al., 2022). PBPK models have already been developed for the chronic kidney disease (CKD) and pediatric populations, as demonstrated in previous literature (Templeton et al., 2018, Franchetti and Nolin, 2020).

The PK of cefepime are altered in various disease states, such as CKD, leading to variations in its elimination and distribution among individuals (Lam and Gomolin 2006). Individuals who suffer from CKD usually have very complicated treatment plans and a significant number of medications to take. The use of multiple drugs and the influence of kidney disease on PK can lead to greater likelihood of adverse events related to use the of drug (Butrovich et al., 2022). The PK of cefepime can be impacted by changes in albumin and HCT levels in patients with renal impairment, making dosing difficult. This modeling approach provides a comprehensive and individualized representation of drug distribution and CL within the body, which can aid healthcare providers in developing tailored dosing regimens for cefepime in patients with renal impairment (Espié et al., 2009, Reynolds and Lefebvre, 2013). The World Health Organization (WHO) categorizes individuals with a body mass index (BMI) less than 25 kg/m^2^ as overweight and those with a BMI greater than 30 kg/m^2^ as obese (Fruh, 2017, Franchetti and Nolin, 2020). Obesity is a chronic medical condition that is related with a range of endocrine, metabolic, cardiovascular, and gastrointestinal disorders, etc. In contrast, nonobese individuals are less prone to infections, whereas obese individuals tend to be more susceptible to bacterial and other types of infections. This is often due to the failure of antibiotic treatment in these patients (Morrison et al., 2021).

The reasoning to create a novel PBPK model for cefepime is that previously no such PBPK model for cefepime has been developed that incorporates the pathophysiological changes occurring in CKD,obese and pediatrics population.The PK profile of cefepime is complex, and the constructed PBPK model can take into account the multiple physiological changes that occur in CKD and obese individuals. For the pediatric population, the model includes age-related variations in blood flow to different organs and can be used to predict absorption, distribution, metabolism and elimination (ADME) characteristics. The objective of this study is to build a PBPK model for cefepime by using a methodical model-building approach that is able to characterize its PK in healthy, disease such as CKD, obese and pediatric populations by integrating specific physiological modifications.

## 2 Materials and methods

### 2.1 Clinical pharmacokinetic data

A total of eight PK studies were included in the PBPK model development process, with five studies in healthy individuals, one in obese individuals, one in individuals with kidney impairment, and one in the pediatric population. One third (2 IV) and two third (7 IV) studies were used in the development and verification of PPBK model respectively and all of them were eventually used in model evaluation. Table 1 provides a summary of the research studies used for PBPK model development and validation.

**Table 1:** Characteristics of the studies chosen for PBPK modeling.

**Sr No.**	**Population**	**No. of Subjects**	**Dose**	**Route of Administrtaion**	**No. of Females**	**Age (years)**	**Weight (kg)**	**Reference**
**Healthy**								
1	Healthy	12	1000 mg	IV Infusion ^a^	6	25.2–27.8	66.5–80.3	(Bächer et al., 1992)
2	Healthy	24	62.5 mg, 125 mg, 250 mg, 500 mg, 1000 mg, 2000 mg	IV Infusion ^a^	0	28.5–33.5	84.6–93.2	(Barbhaiya et al., 1990)
3	Healthy	16	2000 mg	IV Infusion ^b^	0	21–38	N/R	(Garrelts and Wagner 1999)
4	Healthy	6	2000 mg	IV Infusion ^a^	0	23–43	72.2–110	(Nye et al., 1989)
5	Healthy	31	250 mg, 500 mg, 1000 mg, 2000 mg	IV Infusion ^a^	0	20–44	70.6–79	(Barbhaiya et al., 1992)
6	Healthy	48	1000 mg	IV Infusion ^a^	24	20–81	54–86	(Barbhaiya et al., 1992)
**Special Population**								
7	Pediatrics	37	50 mg/kg	IV Infusion ^a^	16	0.175–16.4	3.5–75	(Reed et al., 1997)
**Diseased**								
8	Moderate renal impairment	5	1000 mg	IV Infusion ^a^	0	31.4–60.6	57.5–74.4	(Barbhaiya et al., 1990)
9	Severe renal impairment	5	1000 mg	IV Infusion ^a^	0	27.4–62.6	64.6–101.4	(Barbhaiya et al., 1990)
10	Obese	10	2000 mg	IV Infusion ^a^	5	31–74	106.8–163.2	(Rich et al., 2012)

IV: Intravenous Infusion, N/R: Not reported, ^a^ IV infusion duration 30 min, ^b^ IV infusion duration 3 min, 5 min, 10 min, 15 min.

IV: Intravenous Infusion, N/R: Not reported, ^a^ IV infusion duration 30 min, ^b^ IV infusion duration 3 min, 5 min, 10 min, 15 min.

### 2.2 Strategy for model building

**Fig. 1:**
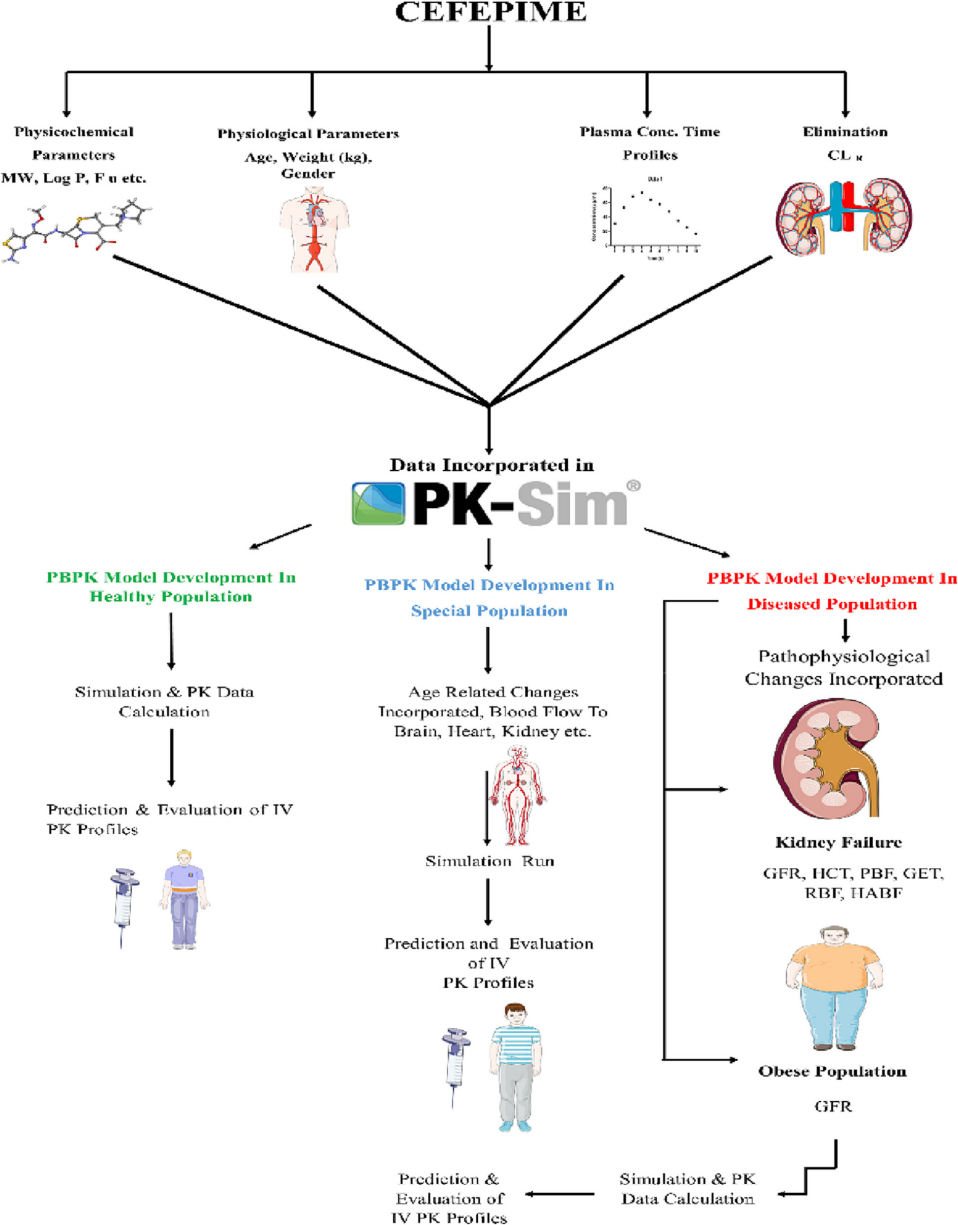
The adopted strategy for the cefepime PBPK model. RBF renal blood flow, PBF plasma protein binding factor, HABF hepatic arterial blood flow, f_u_ fraction unbound, PK pharmacokinetics, MW molecular weight, GET gastric emptying time, IV intravenous, GFR glomerular filtration rate, CL_R_ renal clearance, hematocrit HCT, log P lipophilicity. The figure was created using “Servier Medical Art” https://www.smart.servier.com.

To create the PBPK model of cefepime in various populations (healthy, diseased, and special), an extensive literature search was conducted initially through which studies were selected for the development of the PBPK model (Upton et al., 2016). Cefepime disposition behavior was initially modeled in healthy after the IV administration, and then building blocks were created. Following that, parameters associated with the drug and disease were integrated, and simulations were performed. For each specific study, 100 virtual subjects were generated using the reported demographic data, and simulations were run for each study. The strategy adopted for PBPK model development is described below in Fig. 1.

### 2.3 Modeling software

The PK-SIM® Version 11.0.150 was utilized to generate a PBPK model of cefepime in the special, disease-affected, and healthy population. This software incorporates building blocks, which are organized into seven categories: compound, individual, administration protocols, population, observed data, events, and formulation. The GetData Graph digitizer version 2.26.0.20 was used to scan plasma concentration–time profiles to extract relevant data from the selected studies.

### 2.4 Building blocks

PK-SIM® incorporates building blocks, which are organized into seven categories: compound, individual, administration protocols, population, observed data, events, and formulation. Firstly, information on disease-related factors like plasma protein levels and HCT and data related to cefepime, such as their physical and chemical properties, were obtained through literature searches and added to the building blocks. Then, data points obtained from plasma concentration–time graphs from the included research studies were analyzed using non-compartmental analysis (NCA) using PK-Solver, a Microsoft Excel add-in program (Zhang et al., 2010).

### 2.5 Model parameters

The necessary drug-related input parameters for constructing the cefepime PBPK model are listed in Table 2.The molecular weight of cefepime (C_19_H_24_N_6_O_5_S_2_) is 517 g/mol, and its log P value is 0.69 log units whereas the value of specific clearance obtained from the literature search was 2.2 mL/min/kg and it was optimized to 2.1 mL/min/kg based on visual predictive checks. Moreover, regarding the hepatic clearance, no evidence was available in the literature so only CL_R_ was integrated for the development of the PBPK model. Moreover, the value of f_u_ was optimized to 55%, with a range of (51.6–99.2 %). The Rodgers and Rowland and PK-Sim standard models were used to determine the tissue-plasma partition coefficient and cellular permeability.

**Table 2:** Drug-related input parameters used for the construction of the PBPK model.

**Drug Parameters**	**Value**	**Reference**
**Distribution**		
Partition coefficient model	Rodgers and Rowland	
Fraction unbound %	55 % (51.6–99.2)	(Al-Shaer et al., 2020)
Cellular permeability	Pk–Sim standard	
**Elimination**		
Renal clearance (CL_R_) (ml/min/kg)	2.1 ^a^	(Obach et al., 2008)
**Physicochemical parameters**		
Lipophilicity (log units)	0.6914	(AboulMagd et al., 2021)
pKa 1	3.2	(AboulMagd et al., 2021)
Molecular Weight (g/mol)	517	(hydrochloride)
pKa 2	4.06	(AboulMagd et al., 2021)

a:Value optimized to 2.1 mL/min/kg for model development.

### 2.6 Model structure in pediatric population

The special populations include pregnant women and pediatric patients of different age groups, such as neonates, infants, and children (Rowland Yeo et al., 2011). The pediatric population was given special attention in the development of PBPK model for the special population due to the consideration of different age-related changes in weight, height and other physiological changes. A study was chosen that includes patients between 2 months and 18 years of age (Kim et al., 2010). There have been previously reported changes in blood flow to various organs such as the large intestine, heart, liver, spleen, skin, small intestine, stomach, pancreas, muscle, kidney, and brain, which have been taken into account in the model development i.e., 0.43, 0.35, 0.53, 0.29, 0.23, 1.4, 0.10, 0.08, 0.88, 1.4 and 1.85 L/min, respectively (Edginton et al., 2006).

### 2.7 Model structure in diseased population

#### 2.7.1 Chronic kidney disease (CKD)

PBPK modeling has proven to be an effective method for dose optimization in patients with CKD (Franchetti and Nolin 2020). In the study used for developing the PBPK model in CKD patients, kidney disease was classified into two groups based on CL: moderate renal impairment with the CL range of (31–60 mL/min) and severe renal impairment with (11–30 mL/min) (Barbhaiya et al., 1990). The PBPK model for moderate renal impairment was developed by integrating variations to gastric emptying time (GET), plasma protein binding factor (PBF) and hematocrit (HCT) with values of 20.625 min, 0.9265 and 0.433, respectively (Rowland Yeo et al., 2011). For patients with severe renal impairment, the values of GET, HCT, renal blood flow (RBF), PBF, and hepatic arterial blood flow (HABF) were adjusted and integrated into the cefepime PBPK model with values of 24.375 min, 0.398, 0.837, 0.17 L per minute, and 0.16 L per minute, respectively (Rowland Yeo et al., 2011, Willmann et al., 2021).

#### 2.7.2 Obese population

During the development of a PBPK model for the obese individuals, changes such as a reduced glomerular filtration rate (GFR) of 143 mL/min/1.73 m^2^ from a selected study were incorporated (Rich et al., 2012), the body surface area (BSA) calculated via Du Bois formula or equation (Du Bois and Du Bois 1989). Whereas, other disease-related parameters such as plasma protein albumin and HCT levels remain unchanged in obese and morbidly obese populations (Ghobadi et al., 2011).

### 2.8 Model verification

The development of the cefepime PBPK model was followed by a visual inspection evaluation. This involved comparing the observed data points with the simulated PK data profile. Additionally, the actual values of various PK parameters were compared with the simulated arithmetic means, 5th percentile lower and 95th percentile upper ranges, as well as minimum and maximum plasma concentrations over time profiles. Following that, NCA was performed to compare the observed data values and simulated values of CL, C_max_, and area under the curve from time 0 extrapolated to infinite time (AUC _0–∞_). Equations (1), (2) (Rasool et al., 2021) were used to compute the average fold error (AFE) and the observed to predicted ratio (R_observed_/R_predicted_) for the aforementioned PK parameters.

(1) $$R\;=\frac{\mathrm{Observed}\;value\;of\;PK\;parameter}{\mathrm{Predicted}\;value\;of\;PK\;parameter}$$

(2) $$\;\;\;\;\;\;AFE=\;10^{\frac{\sum \log(fold\;error)}{N}}$$

## 3 Results

### 3.1 PBPK model in healthy population

**Fig. 2:**
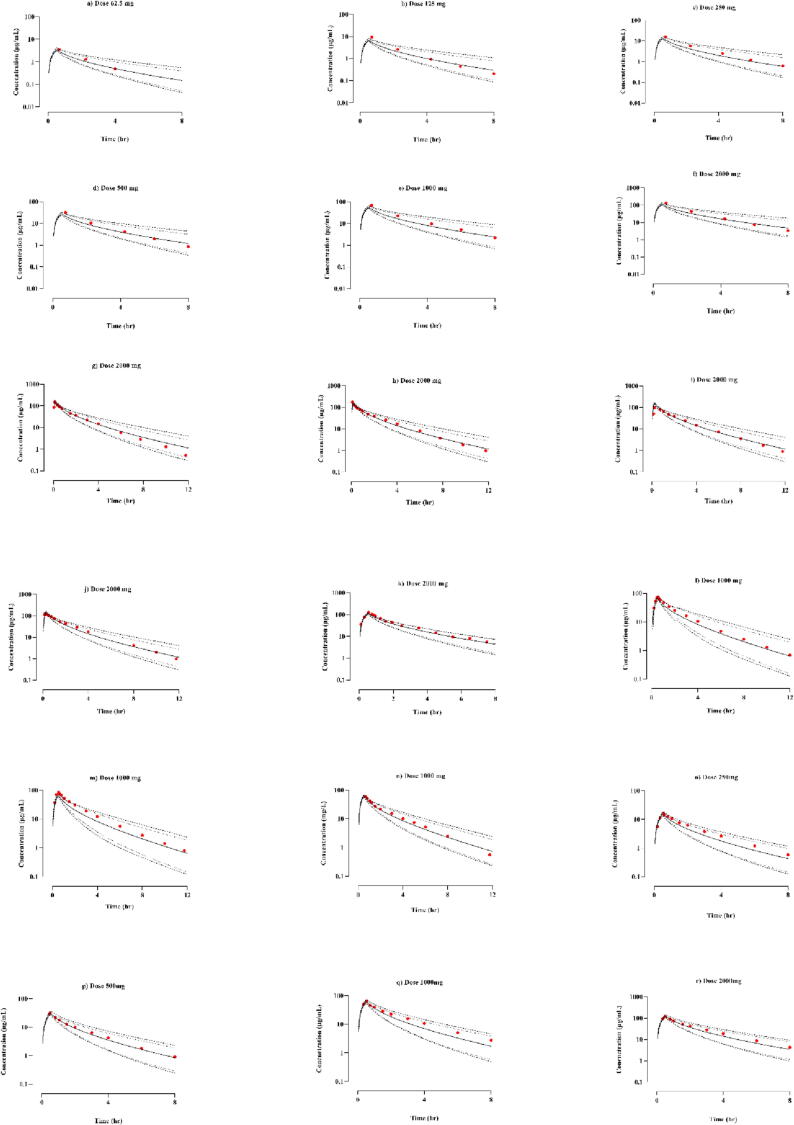
Following the IV infusion of cefepime in healthy subjects at dose range 62.5 mg–2000 mg (a–r) (Nye et al., 1989, Barbhaiya et al., 1990, Bächer et al., 1992, Barbhaiya et al., 1992, Barbhaiya et al., 1992, Garrelts and Wagner, 1999) the comparison of observed and simulated cefepime plasma-concentration versus time graph. The red color dots represents the mean observed data, while the simulated results are shown as the mean solid line, the minimum and maximum values are indicated by dashed lines, the lower 5th percentile is depicted by dotted lines, and the scattered line represents the 95th percentile.

The observed and simulated systemic cefepime concentration profiles following IV dose administration of 62.5–2000 mg to healthy adults is depicted in Fig. 2. The observed data points accurately forecasted the PK of cefepime following IV infusion at the earlier-mentioned dose range, as shown by visual inspection. The mean R_observed_/R_predicted_ values of C_max_ and CL were, 1.04 (95% CI: 0.89–1.19) and 0.84 (95% CI: 0.78–0.89) respectively. Additionally, the AFE was computed for PK parameters, with values for AUC_0–∞_ and C_max_ of 1.20 and 1.04, respectively that were found to be within 2-fold error range thus auspiciously validating the developed model of cefipime. Table 3 displays the R_observed_/R_predicted_ for every study included.

**Table 3:** R_observed/_R_predicted_ values for PK parameters in healthy subjects following the cefepime IV administration.

**PK parameters**	**Healthy**			**References**
	**Obs Value**	**Pred Value**	**R_observed_/R_predicted_**	
**AUC _0-∞_ (μg/mL.h)**				
2000 mg	253.2	224.0	1.13	(Nye et al., 1989)
2000 mg	250.0	218.8	1.14	(Barbhaiya et al., 1992)
2000 mg	271.3	203.4	1.33	(Barbhaiya et al., 1990)
2000 mg	221.5	236.1	0.93	(Garrelts and Wagner 1999)
2000 mg	264.7	236.1	1.12	(Garrelts and Wagner 1999)
2000 mg	219.6	236.0	0.93	(Garrelts and Wagner 1999)
2000 mg	248.0	236.1	1.05	(Garrelts and Wagner 1999)
1000 mg	140.2	109.6	1.27	(Barbhaiya et al., 1990)
1000 mg	150.6	123.0	1.22	(Barbhaiya et al., 1992)
1000 mg	147.0	103.6	1.41	(Barbhaiya et al., 1990)
1000 mg	128.6	113.6	1.13	(Bächer et al., 1992)
1000 mg	173.8	123.0	1.41	(Barbhaiya et al., 1992)
500 mg	58.6	54.1	1.08	(Barbhaiya et al., 1992)
500 mg	66.5	51.8	1.28	(Barbhaiya et al., 1990)
250 mg	34.5	27.3	1.26	(Barbhaiya et al., 1992)
250 mg	36.4	25.9	1.40	(Barbhaiya et al., 1990)
125 mg	17.9	13.0	1.37	(Barbhaiya et al., 1990)
62.5 mg	7.3	6.3	1.15	(Barbhaiya et al., 1990)
**C_max_ (μg/mL)**				
2000 mg	126.3	111.3	1.13	(Nye et al., 1989)
2000 mg	118.1	122.3	0.96	(Barbhaiya et al., 1992)
2000 mg	129.4	85.2	1.51	(Barbhaiya et al., 1990)
2000 mg	150.6	161.8	0.93	(Garrelts and Wagner 1999)

2000 mg	173.1	158.6	1.09	(Garrelts and Wagner 1999)
2000 mg	99.1	154.7	0.64	(Garrelts and Wagner 1999)
2000 mg	128.4	145.0	0.88	(Garrelts and Wagner 1999)
1000 mg	7.1	9.1	0.78	(Barbhaiya et al., 1990)
1000 mg	6.6	8.1	0.81	(Barbhaiya et al., 1992)
1000 mg	6.8	9.6	0.70	(Barbhaiya et al., 1990)
1000 mg	7.8	8.8	0.88	(Bächer et al., 1992)
1000 mg	5.8	8.1	0.71	(Barbhaiya et al., 1992)
500 mg	32.0	30.6	1.06	(Barbhaiya et al., 1992)
500 mg	31.7	22.4	1.41	(Barbhaiya et al., 1990)
250 mg	16.8	15.3	1.09	(Barbhaiya et al., 1992)
250 mg	15.8	11.2	1.41	(Barbhaiya et al., 1990)
125 mg	9.3	5.6	1.66	(Barbhaiya et al., 1990)
62.5 mg	3.5	3.0	1.16	(Barbhaiya et al., 1990)
**CL (L/h)**				
2000 mg	7.9	8.9	0.88	(Nye et al., 1989)
2000 mg	8.0	9.1	0.87	(Barbhaiya et al., 1992)
2000 mg	7.4	9.8	0.75	(Barbhaiya et al., 1990)
2000 mg	9.0	8.5	1.05	(Garrelts and Wagner 1999)
2000 mg	8.1	8.5	0.95	(Garrelts and Wagner 1999)
2000 mg	9.1	8.5	1.07	(Garrelts and Wagner 1999)
2000 mg	7.6	8.5	0.89	(Garrelts and Wagner 1999)
1000 mg	7.1	9.1	0.78	(Barbhaiya et al., 1990)
1000 mg	6.6	8.1	0.81	(Barbhaiya et al., 1992)
1000 mg	6.8	9.6	0.70	(Barbhaiya et al., 1990)
1000 mg	7.8	8.8	0.88	(Bächer et al., 1992)
1000 mg	5.8	8.1	0.71	(Barbhaiya et al., 1992)
500 mg	8.5	9.2	0.92	(Barbhaiya et al., 1992)
500 mg	7.5	9.6	0.78	(Barbhaiya et al., 1990)
250 mg	7.2	9.1	0.79	(Barbhaiya et al., 1992)
250 mg	6.9	9.6	0.71	(Barbhaiya et al., 1990)
125 mg	7.0	9.6	0.72	(Barbhaiya et al., 1990)
62.5 mg	8.6	9.9	0.86	(Barbhaiya et al., 1990)

C_max_: Maximum plasma concentration, Obs: Observed, AUC _0–∞_: Area under the curve from time 0 extrapolated to infinite time, Pred: Predicted, CL: Clearance.

### 3.2 PBPK model in pediatric population

**Fig. 3:**
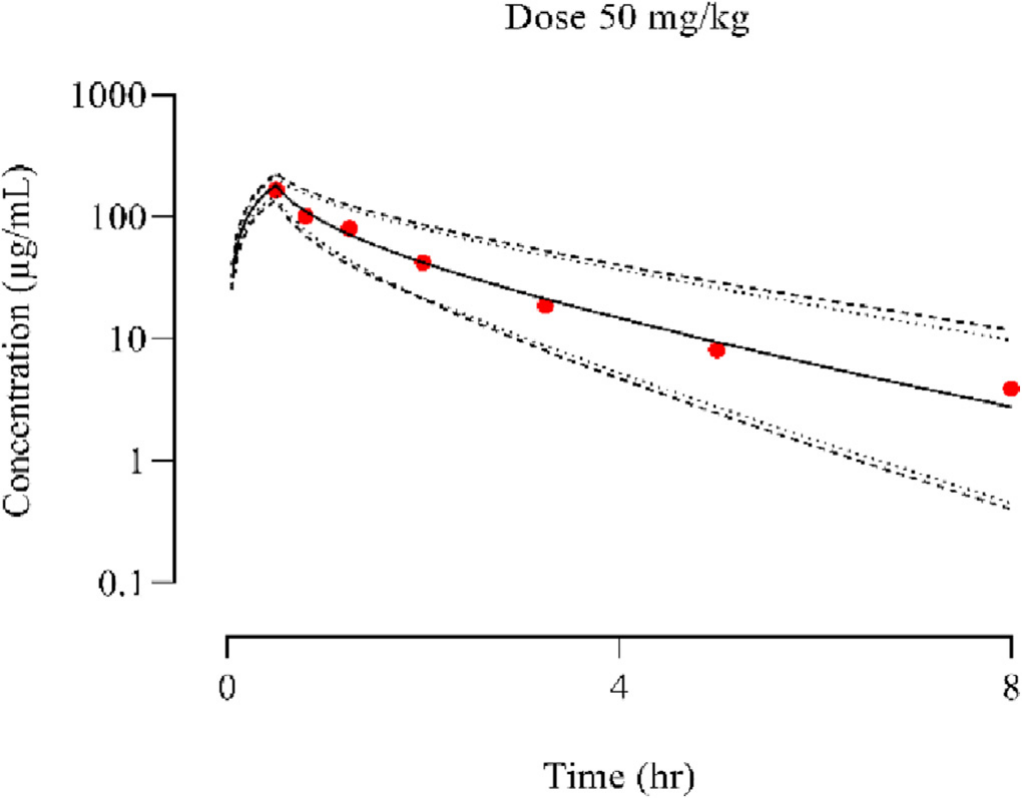
Following the IV infusion of cefepime in pediatrics population at dose of (50 mg/kg)(Reed et al., 1997) the graph above compares observed and simulated plasma concentration. The red color dots represents the mean observed data, while the simulated results are shown as the mean solid line, the minimum and maximum values are indicated by dashed lines, the lower 5th percentile is depicted by dotted lines, and the scattered line represents the 95th percentile.

The Fig. 3 illustrates the comparison between the observed and simulated plasma concentration of cefepime in the pediatric population following a dose of 50 mg/kg. The R_observed_/R_predicted_ values for the CL, AUC_0–∞_, and C_max_ were determined that were 0.9, 0.89 & 1.01 falling within the 2-fold error range (as shown in Table 4).

**Table 4:** R_observed/_R_predicted_ values for PK parameters in pediatric subjects following the cefepime IV administration.

**PK parameters**	**Pediatric subjects**			**Reference**
	**Obs Value**	**Pred Value**	**R_observed_/R_predicted_**	
**Pediatrics**				
**CL (L/h/kg)**	0.18	0.20	0.9	(Reed et al., 1997)
**C_max_ (μg/mL)**	164.45	182.78	0.89	(Reed et al., 1997)
**AUC _0-∞_ (μg/mL.h)**	259.49	255.56	1.01	(Reed et al., 1997)

C_max_: Maximum plasma concentration, Obs: Observed, AUC _0–∞_: Area under the curve from time 0 extrapolated to infinite time, Pred: Predicted CL: Clearance.

### 3.3 PBPK model in disease population

#### 3.3.1 Chronic kidney disease

**Fig. 4:**
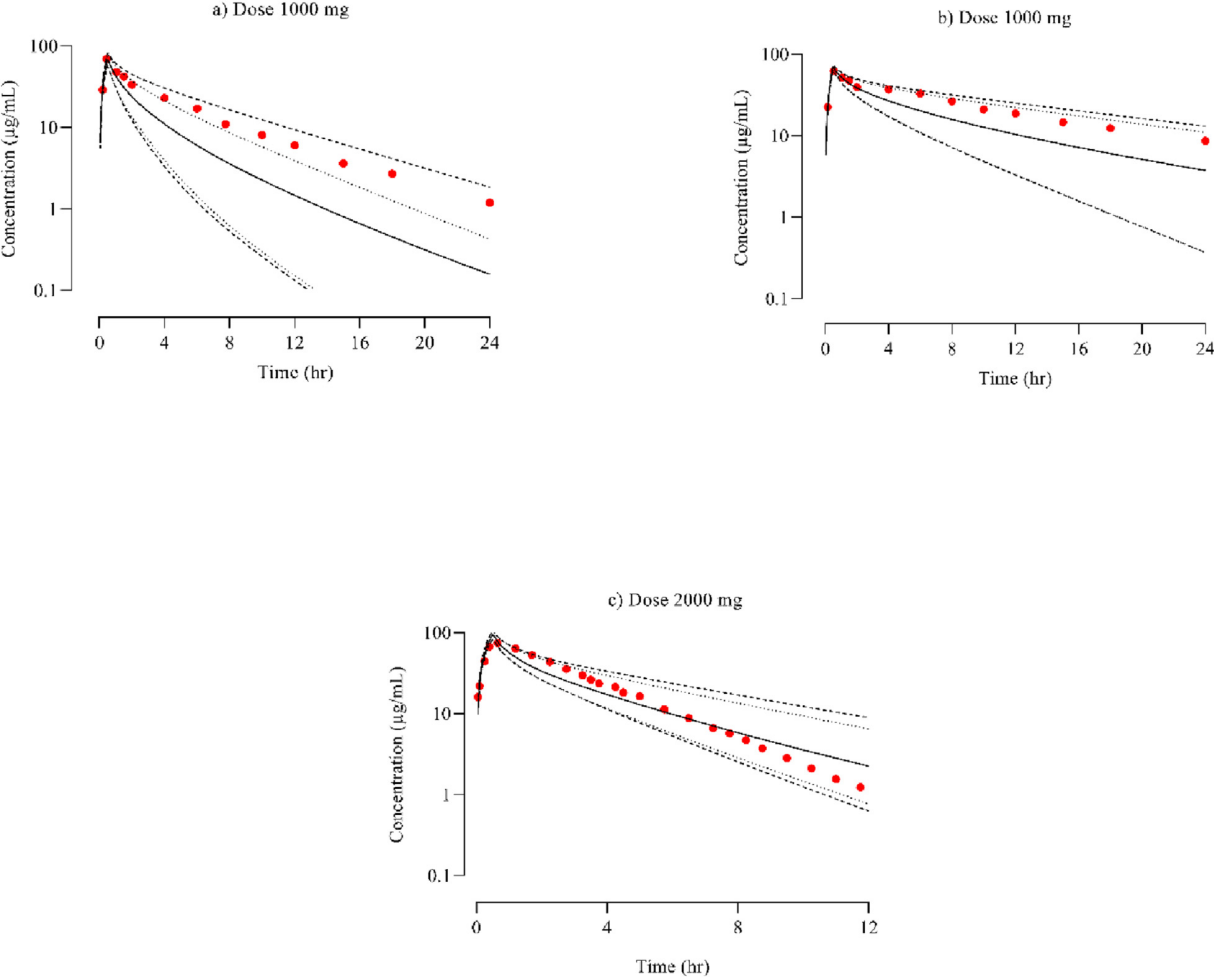
Following the IV infusion of cefepime in CKD population at dose of 1000 mg 7(a) (Barbhaiya et al., 1990) moderate renal impairment 7(b) (Barbhaiya et al., 1990) severe renal impairment and 2000 mg (c) (Rich et al., 2012) in case of obese the graph above compares observed and simulated cefepime plasma concentration. The red color dots represents the mean observed data, while the simulated results are shown as the mean solid line, the minimum and maximum values are indicated by dashed lines, the lower 5th percentile is depicted by dotted lines, and the scattered line represents the 95th percentile.

The Fig. 4 shows the comparison between the actual cefepime concentrations measured in patients with moderate to severe renal impairment and the predicted systemic cefepime concentrations after an IV infusion dose of 1000 mg. The mean R_observed_/R_predicted_ ratios of CL, and C_max_ were 0.6, 0.52 & 0.92, 1.02 in patients of severe and moderate renal impairment respectively as shown in Table 5. The results indicated that all observed to the predicted ratio for PK parameters were within a 2-fold range error.(See Table 7).

**Table 5:** R_observed/_R_predicted_ values for PK parameters in CKD patients following the cefepime IV administration.

**PK parameters**	**Disease**			**Reference**
	**Obs Value**	**Pred Value**	**R_observed_/R_predicted_**	
**Severe Renal Impairment**				
**C_max_ (μg/mL)**	62.55	68.14	0.91	(Barbhaiya et al., 1990)
**CL (L/h)**	1.46	2.4	0.6	(Barbhaiya et al., 1990)
**AUC_0-∞_ (μg/mL.h)**	684.56	415.32	1.64	(Barbhaiya et al., 1990)
**Moderate Renal Impairment**				
**C_max_ (μg/mL)**	69.1	67.3	1.02	(Barbhaiya et al., 1990)
**CL (L/h)**	3.44	6.57	0.52	(Barbhaiya et al., 1990)
**AUC_0-∞_ (μg/mL.h)**	290.109	152.14	1.9	(Barbhaiya et al., 1990)

C_max_: Maximum plasma concentration, Obs: Observed, AUC _0–∞_: Area under the curve from time 0 extrapolated to infinite time Pred: Predicted, CL: Clearance.

**Table 7:** Mean R_observed/_R_predicted_ and AFE values for PK parameters in healthy and renal impairment subjects following the cefepime IV administration.

**PK Parameters**	**R_observed/_R_predicted_**	**Average Fold Error**
**Healthy Subjects**		
**CL**	0.84	0.84
**C_max_**	1.04	1.04
**AUC _0–∞_**	1.21	1.20
**Renal impaired Subjects**		
**CL**	0.56	0.56
**C_max_**	0.96	0.96
**AUC _0–∞_**	1.77	1.76

C_max_: Maximum plasma concentration, AUC _0–∞_: Area under the curve from time 0 extrapolated to infinite time, CL: Clearance.

#### 3.3.2 Obese patients

The PBPK model developed successfully forecasts the systemic cefepime concentration following an IV dose of 2000 mg in obese individuals. The R_observed_/R_predicted_ values for the PK parameters CL and C_max_ were calculated and found to be 0.89 and 0.79 , respectively. Fig. 4 & Table 6 displays the comparison of observed and predicted cefepime concentrations. A visual inspection reveals that the observed concentration falls within the range of the 5th to 95th percentile of the predicted concentration thus showing that cefipime model is accurately developed and validated.

**Table 6:** R_observed/_R_predicted_ values for PK parameters in obese individuals following the cefepime IV administration.

**PK parameters**	**Disease**			**Reference**
	**Obs value**	**Pred value**	**R_observed_/R_predicted_**	
**Obese Individuals**				
**CL (L/h)**	8.21	9.16	0.89	(Rich et al., 2012)
**C_max_ (μg/mL)**	75.98	94.80	0.80	(Rich et al., 2012)
**AUC_0-∞_ (μg/mL.h)**	244.69	220.39	1.10	(Rich et al., 2012)

C_max_: Maximum plasma concentration, Obs: Observed, AUC _0–∞_: Area under the curve from time 0 extrapolated to infinite time, Pred: Predicted, CL: Clearance.

## 4 Discussion

In the study, a whole–body physiologically based pharmacokinetic (WB-PBPK) model was developed to estimate the PK of cefepime in a healthy population. The model was constructed using available physiological and pharmacological data, and was validated using clinical data. The model was then augmented to include patients with CKD, obesity, and special populations by including literature-reported physiological modifications. The resulting model effectively showed the PK of cefepime following IV administration in healthy adult individuals. This reason is supported by a comparison of the mean observed and simulated C_max_ values 59.34 μg/mL (95% CI: 28.57–90.11) and 59.48 μg/mL (95% CI: 27.70–91.26), respectively (Nye et al., 1989, Barbhaiya et al., 1990, Bächer et al., 1992, Barbhaiya et al., 1992, Barbhaiya et al., 1992, Garrelts and Wagner, 1999). Correspondingly, the mean observed AUC_0–∞_ 149.42 μg/mL.h (95% CI: 102.93–195.91) with mean predicted AUC_0–∞_ 139.87 μg/mL.h (95% CI: 97.41–182.43) (Nye et al., 1989, Barbhaiya et al., 1990, Bächer et al., 1992, Barbhaiya et al., 1992, Barbhaiya et al., 1992, Garrelts and Wagner, 1999)were comparable within the dose range of 2000 mg–62.5 mg which further supports that the established PBPK model has effectively forecast the disposition character of drug.

Cefepime is commonly administered to patients as a 30-minute infusion duration (Pais et al., 2022, Pais et al., 2022). However, recent research has shown that altering the infusion duration can have a significant impact on cefepime PK. In the developed PBPK model this same trend was also seen when the drug was infused for shorter durations such as 3, 5, 10, and 15 min. The predicted C_max_ of the cefepime increased from 144.98 μg/mL to 161.83 μg/mL when infusion duration was decreased from 15 min to 3 min. This increase in value of simulated C_max_ with decreasing the infusion duration is consistent with previous research studies conducted on cefepime. This suggests that shortening the infusion duration of cefepime can lead to an increase in C_max_, which may have implications for dosing and administration strategies for this drug (Garrelts and Wagner 1999).

In pediatric patients, the developed PBPK model accurately predicted cefepime disposition character following the IV administration of a dose 50 mg/kg. The AFE for AUC_0–∞_ and C_max_ were 1.01 and 0.89, respectively, indicating that the developed model correctly predicted cefepime PK in the pediatric population. Different variations in blood flow to body organs were incorporated in pediatric model development that includes the brain, kidney, heart, liver, skin, muscle, pancreas, and small intestine (Edginton et al., 2006). The PK of cefepime differ between pediatric and adult population. Specifically, in pediatric patients, the elimination of cefepime primarily occurs through the renal route, whereas in adults, both renal and non-renal elimination pathways are involved. As a result of this difference in elimination, pediatric patients may show slightly different PK parameters than healthy adults (Pais et al., 2022, Pais et al., 2022). In particular, a minor rise in t_1/2_ and increase in simulated value of C_max_ from 111.25 μg/mL to 182.78 μg/mL was seen in the pediatric population. The slight increase in t_1/2_ can be attributed to the fact that in pediatrics the renal function maturation occurs gradually occurs over a period of time. As a a result, the clearance of cefepime is slower, leading to a longer t_1/2_ of the drug in the body. Similarly, the increase in simulated C_max_ values can be explained by the fact that cefepime is eliminated primarily through the kidneys, and pediatric patients have lower CL_R_ rates than healthy adults. As a result, the drug accumulates more in the body, leading to higher concentrations in the blood (Reed et al., 1997).

Extended-spectrum antibiotics, which are used to treat many different types of infections are primarily eliminated from the body through the kidneys. Therefore, it is crucial to adjust the dose of these antibiotics in patients with kidney dysfunction to ensure that the therapy is both safe and effective. If the dose is not adjusted for patients with kidney dysfunction, it can lead to drug toxicity (Cui et al., 2021). Cefepime is predominantly excreted through the kidneys, with 85% being removed through this route. Due to this, dose adjustment is necessary for patients with CKD to ensure safe and effective therapy (Sampol et al., 2000). Kidney impairment is associated with several physiological changes that increase the severity of disease as the level of renal function declines, such as a decrease in HCT volume this decrease can result in changes in drug distribution, metabolism, and elimination, which can impact drug exposure and efficacy. Additionally, CKD is associated with decreased plasma albumin levels, which can further affect drug binding and distribution in the body. These changes must be occupied into consideration when optimizing the dose of cefepime for patients with CKD (Rowland Yeo et al., 2011, Franchetti and Nolin, 2020). Additionally, a decrease in RBF and HABF is observed as the severity of kidney disease advances. The decrease in RBF can result in decreased delivery of drugs to the kidneys, which can affect drug excretion and CL. Similarly, the decrease in HABF can affect drug metabolism in the liver, which can impact drug efficacy and toxicity (Willmann et al., 2021). In the population with CKD, the WB-PBPK model was modified to consider changes such as reduced blood flow to the kidneys and liver to estimate cefepime exposure in these patients. In the developed PBPK model, the physiological parameters such as HCT, GET and PBF significantly reduced when compared to healthy ones and variations in these parameters altered PK of cefepime in moderate renal impaired patients. Whereas the other parameters such as RBF, HABF along with HCT and PBF reduced in severe renal impaired patients however the GET tends to increase in this population and it ultimately leads variation in PK of cefepime (Rowland Yeo et al., 2011). The model precisely portrayed the PK of cefepime in moderate and severe renal impairment and the consideration of decreased blood flow in severe renal impairment significantly enhanced the prediction of cefepime exposure in this patient group. The developed PBPaK model showed rise in the simulated AUC_0–∞_ from 290.109 μg/mL.h to 684.56 μg/mL.h with a progression from moderate to severe renal impairment, which aligns with the results of the chosen research study (Barbhaiya et al., 1990). The primary reason behind the rise in AUC_0–∞_ is believed to be allied with a decline in renal function, which decreases the GFR because the drug stays in bloodstream for approximately 13 h following its administration (Barbhaiya et al., 1990). In another study in patients with damaged kidney, the drug stays in body for approximately 10 h. The decrease in t_1/2_ from previous study was solely due to the fact that the dose used in that study was doubled (Dowell et al., 2022).

Obesity presents a challenge in drug dosing, particularly for obese patients who undergo bariatric surgery, as they are at a high risk of developing postoperative infections if they receive an under-dosed antibiotic. Due to the increased size of organs, blood volume, and blood flow in obese individuals, drug PK may be altered, which affects the required dose to achieve therapeutic concentrations. This highlights the importance of accurate dosing in this population to reduce the risk of postoperative infections (Chopra et al., 2010). The established PBPK model correctly predicted PK of cefepime in obese patients, as the mean R_observed_/R_predicted_ values of PK parameters were inside a 2-fold margin of error. In comparison to healthy participants, the predicted CL in obese individuals increased from 8.9 L/h to 9.1 L/h, which is in line with the observed PK parameter.This in CL can be linked to the rise in blood flow, organ size, and blood volume that is commonly seen in overweight individuals (Rich et al., 2012). These variations eventually affect the required dose of the drug and demonstrate that a greater dose is required to attain the desired antibacterial concentration in obese individuals. **T**he study presents a WB-PBPK model for cefepime in different populations (pediatric, sick, and healthy) and uses data from selected studies to construct and evaluate the model.

The developed PBPK model has few limitations. Firstly no literature or reported value for hepatic CL was available so all CL was assumed to be CLR and integrated into the developed model. Secondly in disease (CKD & Obese) and pediatric populations only a single study was available to validate the developed model that in turn may reduce the significance of results in such populations. Moreover, the obtained PK parameters were consistent with published values, but further research is needed in the field of cefepime PK in infants and children for more accurate results.

## 5 Conclusion

The PBPK model developed for cefepime was found to accurately predict its PK in healthy individuals, as well as those with diseases such as CKD and obesity, and special populations such as pediatrics. The methodical nature of the cefepime PBPK model in renal impaired subjects makes it useful for adjusting doses in patients with kidney impairment. By taking into account age-related changes in organ blood flow in pediatric patients, the model can also predict cefepime exposure in this population. This proposed model has the potential to assist clinicians in optimizing the dose of cefepime in obese individuals.

## Declaration of Competing Interest

The authors declare that they have no known competing financial interests or personal relationships that could have appeared to influence the work reported in this paper.
